# The impact of menstruation persistence or recovery after chemotherapy on survival in young patients with hormone receptor negative breast cancer

**DOI:** 10.1016/j.breast.2020.05.004

**Published:** 2020-05-22

**Authors:** Mark van Barele, Bernadette A.M. Heemskerk-Gerritsen, Helena C. van Doorn, Marjanka K. Schmidt, Maartje J. Hooning, Agnes Jager

**Affiliations:** a Department of Medical Oncology, Erasmus MC Cancer Institute, Rotterdam, the Netherlands; b Department of Gynecological Oncology, Erasmus MC Cancer Institute, Rotterdam, the Netherlands; c Division of Molecular Pathology, The Netherlands Cancer Institute-Antoni van Leeuwenhoek Hospital, Amsterdam, the Netherlands; d Department of Epidemiology, Leiden University, Leiden, the Netherlands

**Keywords:** Menstrual cycle, Hormone receptor-negative breast cancer, Chemotherapy, Cohort study

## Abstract

**Introduction:**

Hormone replacement therapy can diminish hormone depletion-related complaints in postmenopausal women, but is contraindicated for postmenopausal breast cancer (BC) patients. Recovery of menstruation after chemotherapy-induced amenorrhea in young hormone receptor-negative BC patients however, is accepted. To determine the safety of this strategy, we investigated the effect of recovery of menstruation on disease-free survival (DFS) and overall survival (OS) in young hormone receptor-negative BC patients treated with (neo)adjuvant chemotherapy.

**Methods:**

We selected 636 patients from a single-center cohort with early stage hormone receptor-negative BC and under the age of 50 years when treated with chemotherapy. Sufficient data on course of menstruation in medical records was retrospectively found for 397 patients, of whom 299 patients (75%) had a recovery of menstruation after chemotherapy. We used Cox proportional hazards models to estimate hazard ratios (HR) for the effect of recovery of menstruation on DFS and OS.

**Results:**

Patients with recovery of menstruation after chemotherapy less frequently had lymph node involvement at diagnosis (45% vs 66%, p = 0.001). After a median follow-up of 6.7 years, the adjusted hazard ratios were 1.45 (95% CI: 0.83–2.54) for DFS and 1.19 (95% CI: 0.71–1.98) for OS.

**Conclusion:**

No significantly increased recurrence risk was found for hormone receptor-negative BC patients with recovery of menstruation after chemotherapy. However, the outcome of the multivariable model is not reassuring and a potentially increased recurrence risk cannot be excluded. The results need to be validated in a larger prospective study for a more definitive answer.

## Highlights

• Menstruation often recovers after chemotherapy in young breast cancer patients.
• This is currently accepted for those with a hormone receptor negative subtype.
• The cell type such tumors arise from is possibly sensitive to paracrine stimulation.
• Recovery of menstruation may still negatively affect outcome in these patients.

## 1 Introduction

Despite having a reputation of being a disease of the elderly, cancer burden in young adults is quite substantial [1]. Approximately 20% of all invasive breast cancer (BC) patients are younger than 50 years of age at the time of diagnosis [2]. To reduce the risk of recurrent disease, a majority of young patients are treated with chemotherapy [3]. In many of these young patients, menstruation is often suppressed after the first or second cycle of chemotherapy administration and irreversible in a substantial proportion of patients, depending on age and chemotherapy regimen [4,5].

Loss of ovarian activity can have a major impact on quality of life; it results in infertility, often causes climacteric symptoms and increases the risk of osteoporosis and possibly of cardiovascular disease, cognitive impairment and even all-cause mortality [6]. Many of these hormone depletion-related symptoms and risks could theoretically be reduced by hormone replacement therapy (HRT) and interesting new strategies using luteinizing hormone-releasing hormone analogues (LHRH-a) are emerging [7,8]. Of two large randomized trials in patients with stage I to III BC that investigated the risk of HRT on BC recurrence, one study reported on menopausal complaints and indeed showed that HRT led to a decrease in hormone depletion-related symptoms [9]. Importantly though, both studies were terminated prematurely because interim analyses showed an increased risk of BC recurrence in the HRT group [9,10]. Therefore, in general, HRT is contraindicated as treatment of postmenopausal complaints for women with a history of BC. Interestingly, subgroup analyses for estrogen receptor (ER)-negative BC patients showed no significantly increased risk of BC recurrence when treated with HRT (HR 1.9, 95% CI 0.4–9.6 [10] and HR 1.15, 95% CI 0.73–1.80 [9]). However, these analyses were done post-hoc, with relatively small subgroups [10] and no data regarding potentially confounding factors were provided [9]. Therefore, whether there exists a potential risk from HRT use among ER-negative BC patients with complaints of chemotherapy-induced amenorrhea is still a matter of debate.

In clinical practice, recovery of menstruation after chemotherapy-induced amenorrhea in young HR-negative BC patients is fully accepted. Moreover, prescription of HRT to young triple-negative BC (TNBC) patients does happen in some clinics, although data on safety is still lacking [11]. Since the safety of accepting natural recovery of ovarian function in young hormone receptor-negative BC patients treated with (neo)adjuvant chemotherapy is unclear, we investigated the effect of recovery of menstruation on disease-free survival (DFS) and overall survival (OS) in this patient population.

## 2 Methods

### 2.1 Study population

For this single-center consecutive cohort study, we selected BC patients treated in the Erasmus MC Cancer Institute from our institutional cancer registry database using the following inclusion criteria: age at diagnosis <50 years, hormone receptor-negative BC phenotype, BC diagnosed between 1990 and 2014, no evidence of distant metastases at time of diagnosis, and treated with chemotherapy (either neoadjuvant or adjuvant). In accordance with the Dutch breast cancer guidelines, hormone receptor negative phenotype was defined as expression of ER and progesterone receptor (PR) less than 10% in immunohistochemical staining. The HER2-status was defined to be positive if the immunohistochemical staining was 3+, or 2+ with a positive fluorescence in-situ hybridization (FISH) test. HER2 positive BC patients were eligible for inclusion as long as ER and PR were negative.

### 2.2 Data collection

We retrieved data on patient and tumor characteristics, BC treatment and menstrual cycle from the medical files. For the patients in our cohort, reporting on menstrual cycle and menopausal status was not standardized, but performed at the discretion of the physician doing the follow-up. Patients reported to have persisting menstruation during and after chemotherapy, and patients reported to have had recovery of menstruation at any point during follow-up were allocated to the group with recovery and persistence of menstruation, for readability now referred to as the “recovery of menstruation” group. Women experiencing chemotherapy-induced amenorrhea without any recovery during follow-up were allocated to the group without recovery of menstruation after chemotherapy. In the latter group, to prevent misclassification of women who had a delayed recovery of menstruation, an additional confirmation of postmenopausal status at least 12 months after diagnosis was required for those patients younger than 45 years of age at BC diagnosis. For older patients, considering the fact that recovery of menstruation after chemotherapy-induced amenorrhea is very unlikely after 12 months, no additional confirmation was required [12].

### 2.3 Statistical analyses

We tested for differences between the two groups using the chi-squared test for categorical variables and the Wilcoxon rank-sum test for continuous variables. The primary endpoint of the study was disease-free survival (DFS), defined as time at risk until first loco-regional recurrence, distant metastasis, ipsilateral second breast cancer or BC-related death (not preceded by known loco-regional recurrence or distant metastasis). Overall survival (OS), the secondary endpoint, was defined as time at risk until all-cause death. The observation period started one year after BC diagnosis, since recurrent disease within one year after BC diagnosis is unlikely to be attributed to changes in hormonal status. Censoring events for the DFS analyses were a new cancer diagnosis other than ipsilateral BC (excepting incidental FIGO stage I ovarian cancer not requiring chemotherapy, non-melanoma skin cancer and cervical intra-epithelial neoplasia), death not related to BC, and date of last follow-up. For the OS analyses, date of last follow-up was the only censoring event. Due to the retrospective nature of this study, data on course of menstruation was missing for a substantial proportion of the patients. To investigate whether exclusion of these patients has led to selection bias, we compared both in- and excluded patients on the main factors of interest. To explore the effect of different definitions of the DFS endpoint, we performed two sensitivity analyses, one including contralateral second breast cancers and the other excluding ipsilateral and contralateral second breast cancers. An overview of all analyses is displayed in Supplementary Table 1.

We used both stepwise inclusion and full multivariable Cox proportional hazards models to calculate hazard ratios and accompanying 95% confidence intervals (CI) for DFS and OS, using the group without recovery of menstruation as the reference. Age, known *BRCA* mutation (yes/no), tumor size, lymph node status, neoadjuvant chemotherapy (yes/no), endocrine therapy (yes/no), and risk-reducing salpingo-oophorectomy (RRSO) during the observation period (yes/no, time-dependent) were considered as potential confounders. For the stepwise model, we included a potential confounder into the multivariable model if 1) there was a significant difference between the two groups for the variable, 2) the likelihood ratio test showed a significant difference between the models with and without the variable, and 3) there was no significant interaction of the variable with the main variable of interest (i.e. recovery of menstruation yes or no). For each variable, the proportional hazards assumption was inspected visually by drawing a graph of log(-log(survival)) against log(survival time) and tested formally using scaled Schoenfeld residuals.

We used STATA (version15.1, StataCorp, College Station TX, USA) for all analyses. All p-values were two-sided and a significance level α = 0.05 was used.

## 3 Results

### 3.1 Study population

**Fig. 1:**
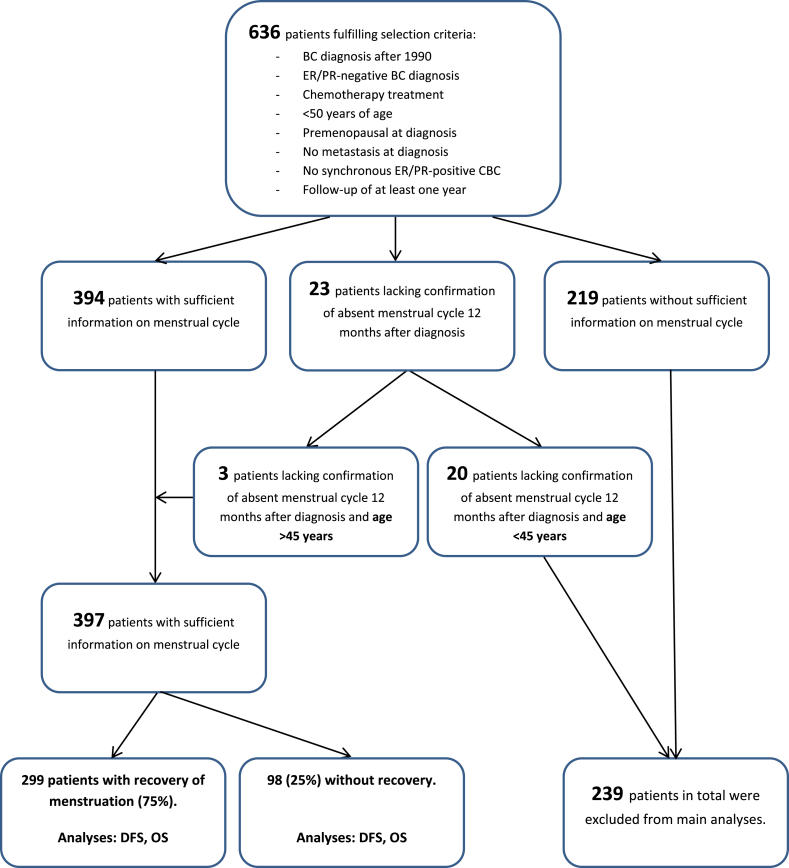
**Flow diagram of patient selection**. Abbreviations: BC, breast cancer; ER, estrogen receptor; PR, progesterone receptor; CBC, contralateral breast cancer; DFS, disease-free survival; OS, overall survival.

The patient selection procedure is summarized in a flow-diagram (Fig. 1). For 397 patients out of 636 (62%), sufficient data on menstrual status was available in the medical files, of whom 299 patients (75%) had recovery of menstruation. Patients with recovery of menstruation after chemotherapy were younger at BC diagnosis than patients without recovery of menstruation (median age of 34.6 vs 43.7, p < 0.001), were more often without lymph node involvement (55% vs 34%, p = 0.001), and were less often treated with endocrine therapy (3% versus 13%, p < 0.001). The proportion of *BRCA*-mutation carriers was high in both groups (48% and 47% in the group with and without recovery, respectively). Patients with recovery of menstruation more often underwent prophylactic mastectomy than patients without recovery (34% vs 20%, p = 0.013), but less often opted for RRSO (27% vs 43%, p = 0.003) (Table 1).

**Table 1:** **Comparison of patient and tumor characteristics between recovery and no recovery of menstruation.** Abbreviations: AI, aromatase-inhibitor; LHRH, luteinizing hormone-releasing hormone; RRM, risk-reducing mastectomy; RRSO, risk-reducing salpingo-oophorectomy; NAC, neoadjuvant chemotherapy; AC, Doxorubicin (Adriamycin) + Cyclophosphamide; CMF, Cyclophosphamide+Methotrexate+Fluorouracil; FAC / FEC, Fluorouracil + Doxorubicin/Epirubicin + Cyclophosphamide.

	Recovery of menstruation after chemotherapy N= 299 (75%)	No recovery of menstruation after chemotherapy N= 98 (25%)	p-value
Follow-up time in years, median (range)	6.2 (1.0 – 23.7)	8.1 (1.1 – 27.0)	0.0260
Age at diagnosis, median years (range)	34.5 (23.6 - 49.6)	43.6 (27.2 – 49.9)	<0.001
Year of diagnosis, median (range)	2004 (1991-2014)	2003 (1990-2013)	0.0182
Year of diagnosis, 5-year categories			0.001
1990-1994	12 (4)	15 (15)	
1995-1999	31 (10)	16 (16)	
2000-2004	113 (38)	26 (27)	
2005-2009	86 (29)	26 (27)	
2010-2014	57 (19)	15 (15)	
Proven *BRCA* mutation			0.743
No	156 (52)	53 (53)	
Yes	143 (48)	45 (47)	
*BRCA1*	134 (45)	37 (38)	
*BRCA2*	9 (3)	7 (8)	
*BRCA1+BRCA2*	0	1 (1)	
Tumor size (pT/cT)^a^			0.592
1	130 (44)	38 (40)	
2	137 (46)	45 (48)	
3	16 (5)	8 (9)	
4	15 (5)	3 (3)	
Unknown	1	4^b^	
Lymph-node status (pN/cN)^a^			0.001
0	163 (55)	33 (34)	
1	88 (29)	41 (42)	
2	31 (10)	10 (10)	
3	17 (6)	14 (14)	
Tumor grade			0.616
<3	28 (10)	7 (9)	
3	250 (90)	78 (92)	
Unknown	21	13	
HER2 status			0.970
HER2+	43 (18)	13 (19)	
HER2-	191 (82)	57 (81)	
Unknown	65	28	
Type of surgery			0.022
No surgery	0	2 (2)	
Lumpectomy	165 (55)	46 (47)	
Mastectomy	134 (45)	50 (51)	
Radiotherapy received	209 (70)	73 (74)	0.572
Endocrine therapy^c^	9 (3)	14 (14)	<0.001
Tamoxifen	45 (44)	9 (75)	
Tamoxifen + AI	1 (11)	0	
Tamoxifen + LHRH-analogue	1 (11)	1 (8)	
LHRH-analogue alone	3 (33)	2 (17)	
Targeted therapy	32 (11)	9 (9)	0.668
RRM	101 (34)	20 (20)	0.013
RRSO	80 (27)	42 (43)	0.003
Chemotherapy	299	98	
Of whom NAC	32 (11)	13 (13)	0.487
Chemotherapy regimen			0.004
AC	110 (37)	28 (29)	
CMF	11 (4)	14 (14)	
FAC / FEC	72 (24)	24 (24)	
Taxane-containing	83 (28)	27 (28)	
Other / Unknown	23 (7)	5 (5)	

Eleven patients were not included in the disease-free survival (DFS) analysis because of a DFS-related event or censoring event before the start of observation (i.e. one year after breast cancer diagnosis). Therefore, 397 patients were eligible for overall survival (OS) analysis and 386 were eligible for DFS analysis.

a For patients who were treated with neoadjuvant chemotherapy, the clinical stage is reported.

b Three out of these four patients with an unknown tumor size were reported to have occult breast cancer, with pathologically proven adenocarcinoma metastasis in the axilla.

c Reasons for patients receiving endocrine therapy were the following: tamoxifen as primary adjuvant treatment as part of the EORTC 10901 trial (n=10); tamoxifen for unknown reasons (n=2); tamoxifen (n=1) and tamoxifen+LHRH-analogue (n=1) as a result of a false positive progesterone receptor test; tamoxifen+AI (n=1) and tamoxifen+LHRH-analogue (n=1) for an ER/PR of 1-9%; LHRH-analogue during chemotherapy to protect the ovaries (n=1); LHRH-analogue to suppress a recovery of menstruation (n=1); LHRH-analogue for unknown reasons (n=2). For two patients, we could not find what specific endocrine therapy was prescribed nor the reason for it.

Comparing baseline characteristics between these included patients (n = 397) and the excluded patients (n = 219) revealed significant differences in median age at diagnosis (36.8 vs 40.0, p < 0.001), HER2 positivity (19% vs 30%, p = 0.007) and complementary treatment with trastuzumab (11% vs 17%, p = 0.018). More patients in the excluded group received radiotherapy (71% vs 87%, p < 0.001) (Supplementary Table S1).

### 3.2 Disease-free survival

Among those patients who developed a BC related event, 22 patients experienced a loco-regional recurrence, 46 patients a distant metastasis, seven patients an ipsilateral second primary breast cancer and one patient died of BC without previous documentation of a recurrence (Table 2).

**Table 2:** **Comparison of disease-free and overall survival endpoints between recovery and no recovery of menstruation groups.** Abbreviations: BC, breast cancer.

Disease-free survival endpoints (main analysis):	Recovery of menstruation	No recovery of menstruation
	N=294	N=92
	Number (%)	Number (%)
Loco-regional recurrence only	17 (6)	5 (5)
Distant metastases	36 (12)	10 (11)
2^nd^ primary ipsilateral BC	6 (2)	1 (1)
BC-related death	1 (0)	0
Overall survival endpoints (main analysis):	Recovery of menstruation	No recovery of menstruation
	N=299	N=98
	Number (%)	Number (%)
Total deaths	61 (20)	22 (22)
Causes:		
Breast cancer	56 (19)	21 (21)
Ovarian cancer	1 (0)	0
Lung cancer	1 (0)	1 (1)
Unknown cause	3 (1)	0

**Fig. 2:**
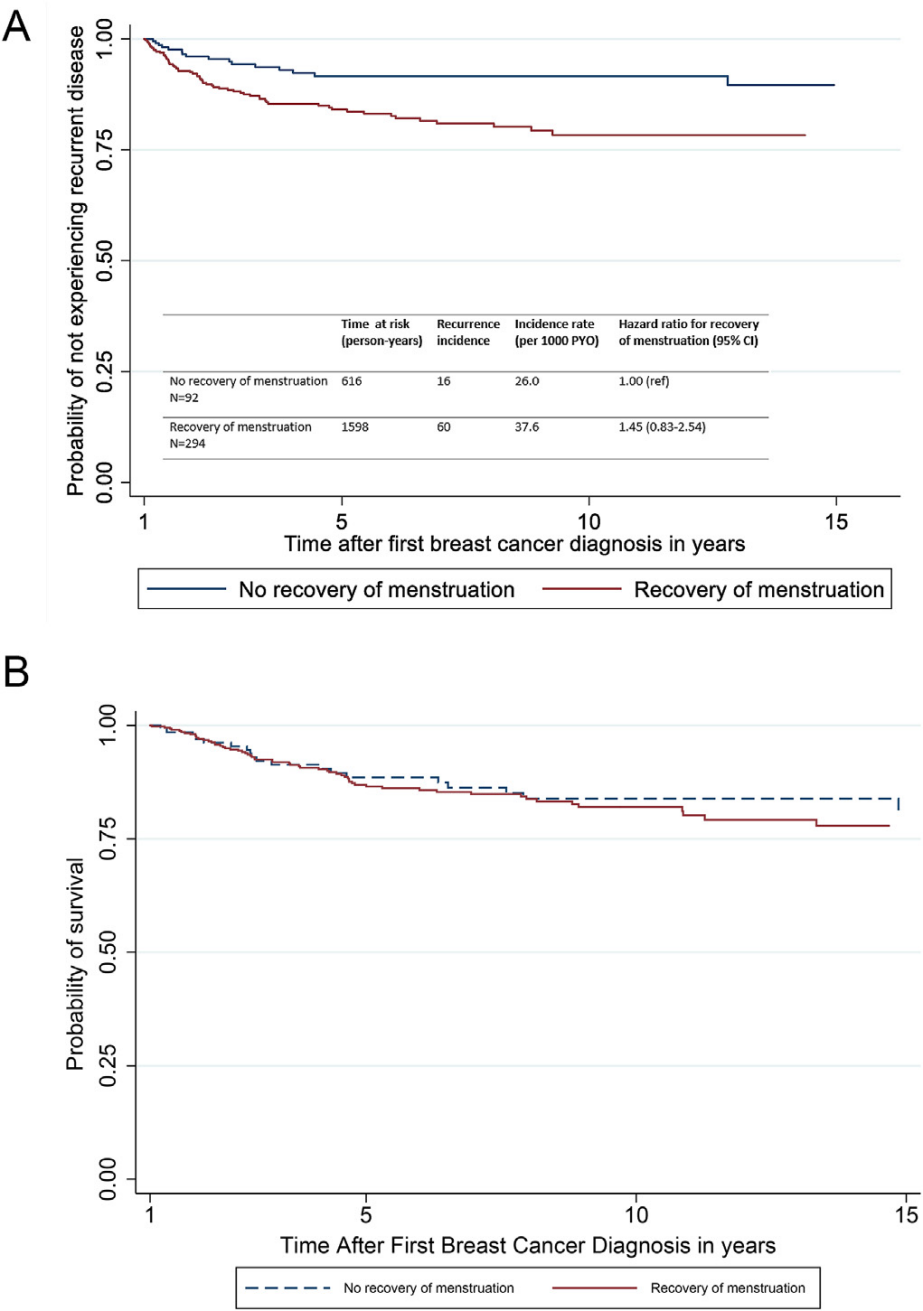
A. Disease-free survival curves based on the cox proportional hazards model, adjusted for lymph node status. B. Overall survival curves based on the cox proportional hazards model, adjusted for lymph node status and endocrine therapy.

Recovery of menstruation after chemotherapy for BC was not associated with an increased risk of recurrent disease in the univariable analysis (hazard ratio 1.24, 95% CI 0.71–2.15, Table 3, Fig. 2A). After adjusting for lymph-node status only (in a stepwise model) or other relevant variables (full multivariable model) the recovery of menstruation was associated with a non-significant higher risk of recurrent disease, showing an HR of 1.45 (95% CI: 0.83–2.54) for the stepwise model and an HR of 1.31 (95% CI: 0.66–2.59) for the full model (Table 3).

**Table 3:** Hazard ratios from univariable and multivariable Cox proportional hazards models for main analyses of DFS and OS. Abbreviations: DFS, disease-free survival; OS, overall survival; HR, hazard ratio; ref, reference group; RRSO, risk-reducing salpingo-oophorectomy.

	DFS			OS		
	Univariable HR (95% CI)	HR in stepwise multivariable model (95% CI)^a^	HR in full multivariable model (95% CI)^‡^	Univariable HR (95% CI)	HR in stepwise multivariable model (95% CI)^a^	HR in full multivariable model (95% CI)^‡^
Recovery vs. no recovery of menstruation	1.24 (0.71–2.15)	1.45 (0.83–2.54)	1.31 (0.66–2.59)	1.07 (0.66–1.76)	1.19 (0.71–1.98)	0.98 (0.51–1.88)
Age at diagnosis, continuous	1.00 (0.97–1.04)		0.99 (0.95–1.04)	1.00 (0.97–1.04)		0.98 (0.93–1.02)
Untested or no *BRCA* mutation	1.00 (ref)		1.00 (ref)	1.00 (ref)		1.00 (ref)
*BRCA1* mutation	0.58 (0.36–0.94)		0.62 (0.36–1.09)	0.43 (0.27–0.69)		0.50 (0.28–0.90)
*BRCA2* mutation	1.11 (0.44–2.79)		1.24 (0.45–3.41)	0.55 (0.17–1.76)		0.72 (0.21–2.47)
Tumor size (pT/cN)						
1	1.00 (ref)		1.00 (ref)	1.00 (ref)		1.00 (ref)
2	2.29 (1.36–3.87)		2.11 (1.24–3.59)	1.77 (1.07–2.94)		1.65 (0.99–2.78)
3	2.57 (1.03–6.36)		1.34 (0.48–3.72)	3.61 (1.72–7.56)		1.99 (0.86–4.59)
4	4.07 (1.64–10.1)		1.89 (0.62–5.81)	4.49 (2.01–10.0)		2.01 (0.73–5.57)
Lymph node status (pN/cN)						
0	1.00 (ref)	1.00 (ref)	1.00 (ref)	1.00 (ref)	1.00 (ref)	1.00 (ref)
1	1.59 (0.94–2.68)	1.66 (0.98–2.80)	1.44 (0.84–2.49)	2.03 (1.21–3.42)	2.11 (1.25–3.56)	1.66 (0.96–2.89)
2	1.73 (0.84–3.57)	1.79 (0.87–3.69)	1.76 (0.83–3.72)	2.32 (1.20–4.50)	2.87 (1.47–5.59)	2.39 (1.20–4.76)
3	2.87 (1.40–5.92)	3.16 (1.52–6.59)	2.83 (1.27–6.27)	2.59 (1.24–5.41)	3.11 (1.47–6.60)	2.25 (1.02–4.94)
Neoadjuvant chemotherapy, yes vs no	2.12 (1.14–3.94)		1.66 (0.77–3.64)	3.10 (1.83–5.25)		1.74 (0.86–3.51)
Endocrine therapy, yes vs no	0.64 (0.20–2.03)		0.55 (0.17–1.82)	0.40 (0.12–1.29)	0.30 (0.09–0.99)	0.28 (0.08–1.00)
RRSO, yes vs no, time-dependent	1.02 (0.48–2.19)		1.63 (0.68–3.90)	0.52 (0.25–1.06)		1.04 (0.45–2.40)

a Multivariable hazard ratio is for the model constructed using the forward stepwise process described in the Methods section. ‡ The full model includes all clinically relevant variables, regardless of statistical significance (age, tumor size, lymph node status, BRCA mutation, RRSO as time-dependent variable, neoadjuvant therapy, endocrine therapy).

### 3.3 Overall survival

The OS analysis included eleven more patients who were censored in the DFS analysis because of a DFS-related, but not OS-related (censoring) event prior to the start of the observation period. Recovery of menstruation after chemotherapy was not associated with OS in univariable analysis (hazard ratio 1.07, 95% CI 0.66–1.76; Table 3, Fig. 2B). After adjusting for lymph-node status and endocrine therapy (in a stepwise model) or other relevant variables (full multivariable model) recovery of menstruation was not associated with a higher risk of death, showing an HR of 1.19 (95% CI: 0.71–1.98) for the stepwise model and an HR of 0.98 (95% CI: 0.51–1.88) for the full model (Table 3).

### 3.4 Additional analyses

Considering the variety of DFS definitions, two sensitivity analyses were performed with differing endpoints for DFS (including or excluding ipsilateral and contralateral second primary breast cancer), in which similar results were found (Supplementary Table 1).

To determine the presence of a potential selection bias, we used Cox proportional hazards models to compare the survival of all included patients with that of the excluded patients. We found similar a DFS for the excluded group (full model HR: 0.88, 95% CI 0.57–1.35), but a trend for worse overall survival for the excluded group (full model HR: 1.35, 95% CI 0.94–1.93).

## 4 Discussion

Our results showed that recovery of menstruation after chemotherapy did not have a significant effect on the risk of recurrent disease or mortality in young patients with hormone receptor-negative BC. While consistent with the idea that, from a clinical viewpoint, hormone exposure should not impact survival in hormone receptor-negative BC, the multivariable models are not particularly reassuring. The hazard ratio of 1.45 found in the multivariable DFS model, although not significant, raises the concern that a deleterious effect of hormone exposure in hormone receptor-negative BC patients cannot be excluded.

Our findings appear to be in contrast with previous studies investigating the impact of recovery of ovarian function after chemotherapy in BC patients, as demonstrated in a meta-analysis by Zhou et al. [13]. In their hormone receptor-negative subgroup analysis of three studies, they show no difference in survival between recovery and absence of ovarian activity after chemotherapy (HR 0.97, 95% CI: 0.66–1.41). One of the most recent studies concerning the prognostic effect of chemotherapy-induced amenorrhea in HR-negative BC patients did not find a difference in survival either [14].

A possible explanation for the discrepancy of our result could be the differences in how hormonal exposures were defined. Most previous studies used a certain period (usually three or six months) of absence of menstruation (amenorrhea) after chemotherapy, to define the absence of hormonal exposure [15]. This definition leads to classifying any woman with menstruation recovering after this specific period being classified as not having hormonal exposure. This misclassification increases the risk in the hormonally non-exposed group if an increased risk from hormone exposure does indeed exist. As a result, this mixing of exposures artificially lowers the risk ratio for hormone exposure (as both groups will now have an increased risk). The only other study using a similar definition for ovarian activity as we did, with a median duration of FU of 6.2 years, was not included in the meta-analysis by Zhou et al., 2015 [13]. This study showed a trend towards worse DFS for the subgroup of hormone receptor-negative BC patients with recovery of menstruation after chemotherapy (HR 1.73, 95% CI 0.86–3.48) [16]. Combining this result with ours in a pooled analysis yields an HR of 1.55 (95% CI 1.00–2.40), suggesting that the statistical non-significance of our primary result may be due to insufficient power (Supplementary Fig. 1). This further strengthens our concern that a potentially increased recurrence risk from hormone exposure by means of recovered menstruation, cannot be excluded.

Our definition of hormone receptor-negative breast cancer using <10% expression of ER and PR on immunohistochemistry, may have affected our results. Previous studies have shown that from a clinical point of view, tumors with 1–9% receptor expression often behave like triple negative tumors as far as endocrine sensitivity is concerned. In these studies, no benefit of adjuvant endocrine therapy in the population with hormone receptor expression between 1 and 9% was found, similar to hormone receptor expression less than 1% [17,18]. Therefore, it is our opinion that the inclusion of patients with 1–9% ER/PR expression cannot explain the relatively high adjusted HRs of e.g. 1.45 for DFS in our study.

There may be biological explanations for a potential risk of hormonal exposure in hormone-receptor negative BC patients. One potential mechanism through which female steroidal hormones can act upon ER/PR-negative (tumor) cells is via Receptor Activator of Nuclear factor Kappa(κ)-B (RANK) and its ligand, RANKL. In short, ER/PR positive benign mammary cells are stimulated to produce RANKL when progesterone binds the PR receptor [19,20]. RANKL in turn, is capable of stimulating proliferation in neighboring ER/PR-negative cancer cells in a paracrine fashion by binding to the RANK receptor on these cancer cells [21,22]. Interestingly, in a study randomizing ER/PR-negative BC patients to either chemotherapy or chemotherapy with goserelin (a LHRH-analogue), those in the goserelin arm had a better overall survival as well as higher rates of recovery of menstruation and pregnancy [23]. A recent meta-analysis of this and several other studies on LHRH-analogue use shows that the survival benefit in ER-negative BC patients largely remains, although no longer statistically significant (HR 0.65; 95% CI 0.39–1.07) [24]. While this is no definitive proof, it illustrates that endocrine effects do appear to be present in hormone receptor-negative BC patients. Another potential mechanism could be endocrine activity via the ERβ receptor, which is not routinely tested for when determining the ER/PR status of a breast tumor. Interestingly, in one study 70% of the 105 ERα-negative patients in the cohort were ERβ-positive (weak and strong immunohistochemistry staining combined) [25], whereas another found ERβ-positivity in as much as 60% of ERα-negative cases [26]. The exact role of ERβ in ER-negative BC however, is not yet completely understood.

To the best of our knowledge, our study is unique in that it is the first to specifically look into the effects of hormone exposure in hormone receptor negative BC, with a fairly large study population. A potential weakness of our study is that data on menstrual cycle status were not always recorded in the medical file, resulting in the exclusion of a large group of patients of whom menopausal status is unknown (34%). Apart from age, *BRCA* mutations and HER2 positivity with complementary targeted treatment, the excluded group did not differ much from our included population when comparing baseline characteristics (Supplementary Table S2). Because the excluded group represents a fairly large proportion of our initial patients, we compared survival of those patients included with survival of those left out of the study, showing a worse overall survival for the excluded patients (Supplementary Fig. 2). Physicians most likely have omitted questions about recovery of menstruation among those patients with an early recurrence due to focus on more important issues to discuss at such moments, leading to a skewed distribution of patients with poor prognosis towards the excluded group. A sensitivity analysis where the excluded patients are added to the group with recovery of menstruation shows a further increase of risk associated with recovery of menstruation. When added to the non-recovery group, no more negative effect of recovery of menstruation on mortality is seen. However, it is unlikely that all these excluded patients would have ended up in the group without recovery of menstruation, as the median age was forty (Supplementary Table S2) and recovery of menstruation is therefore expected in the majority of patients. Furthermore, in the study by Park and colleagues menstrual cycle status was recorded at each visit to the outpatient clinic during follow-up. This prospective gathering of data on menstrual cycle status makes it unlikely that selection bias as described above could have occurred, yet their results are very similar to ours [16].

In conclusion, although recovery of menstruation after chemotherapy did not have a significant effect on the risk of recurrent disease or mortality in young hormone receptor-negative BC patients, the multivariable analyses were not particularly reassuring. Although HRT does not function exactly as endogenous hormones, and risks may therefore differ, we argue that prescribing HRT to hormone receptor-negative BC patients suffering from menopausal complaints should not be considered lightly, and perhaps non-hormonal treatment options should be explored first. Further research on the safety of hormones and/or HRT in young hormone receptor-negative BC patients is clearly warranted.

## Funding source

Funding for this research was provided by the 10.13039/501100010429Erasmus MC Cancer Institute. No external funding was obtained.

## Ethics committee approval

For this retrospective research, no additional ethics committee approval was required as per Dutch law.

## Declaration of competing interest

None of the authors have a conflict of interest to declare regarding the contents of this work.
