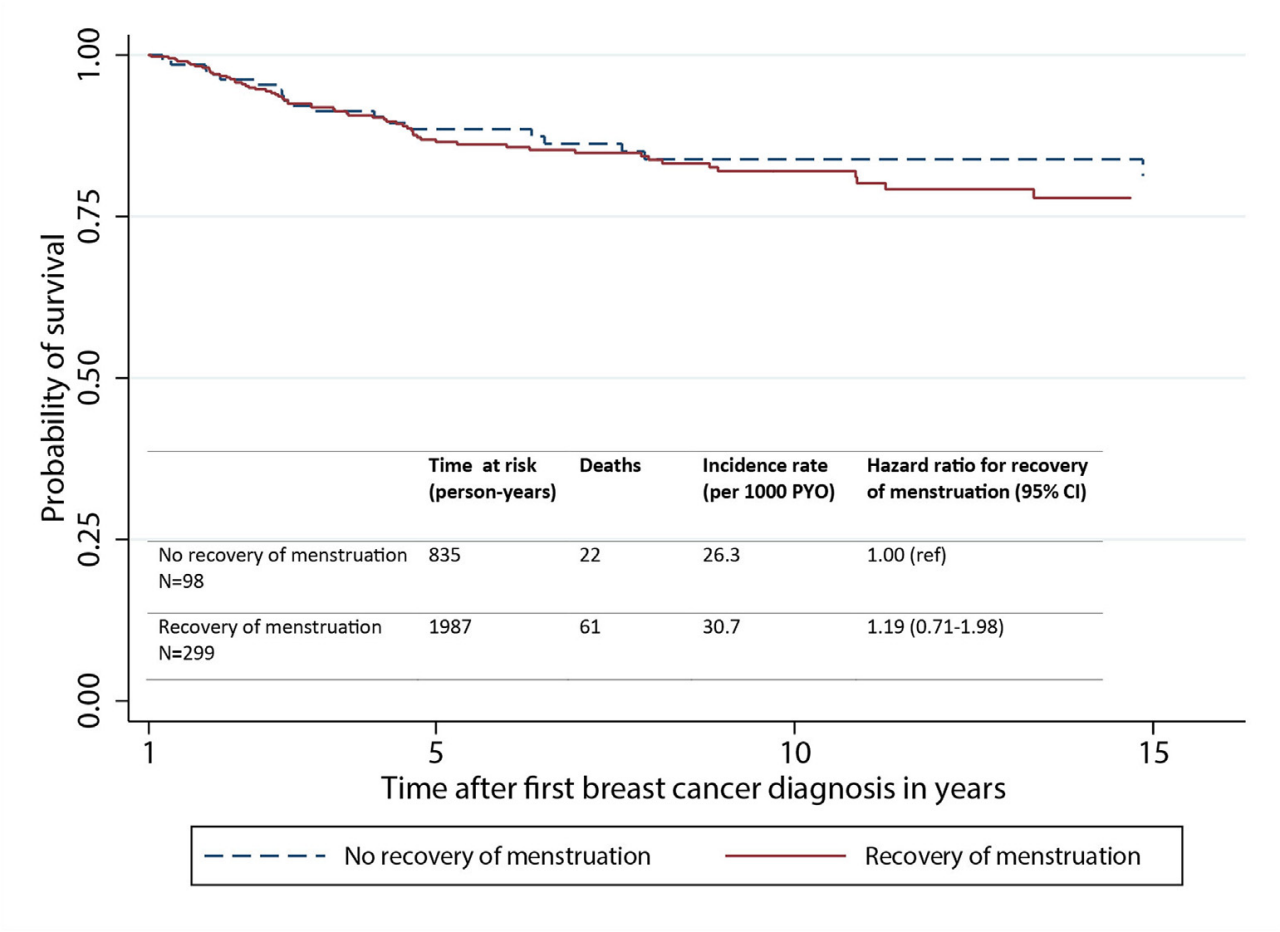# Corrigendum to “The impact of menstruation persistence or recovery after chemotherapy on survival in young patients with hormone receptor negative breast cancer” [Breast 52 (2020) 102–109]

**DOI:** 10.1016/j.breast.2020.08.016

**Published:** 2020-09-16

**Authors:** Mark van Barele, Bernadette A.M. Heemskerk-Gerritsen, Helena C. van Doorn, Marjanka K. Schmidt, Maartje J. Hooning, Agnes Jager

**Affiliations:** a Department of Medical Oncology, Erasmus MC Cancer Institute, Rotterdam, the Netherlands; b Department of Gynecological Oncology, Erasmus MC Cancer Institute, Rotterdam, the Netherlands; c Division of Molecular Pathology, The Netherlands Cancer Institute-Antoni van Leeuwenhoek Hospital, Amsterdam, the Netherlands; d Department of Epidemiology, Leiden University, Leiden, the Netherlands

The authors regret the introduction of two errors in Fig. 2 during the proofing process. The incidence table in Fig. 2B had been omitted and two solid lines instead of one solid and one dotted line were present in Fig. 2A. Corrected versions for both graphs have been provided. The authors would like to apologise for any inconvenience caused.

Fig. 2A. Disease-free survival.

**Figure:**
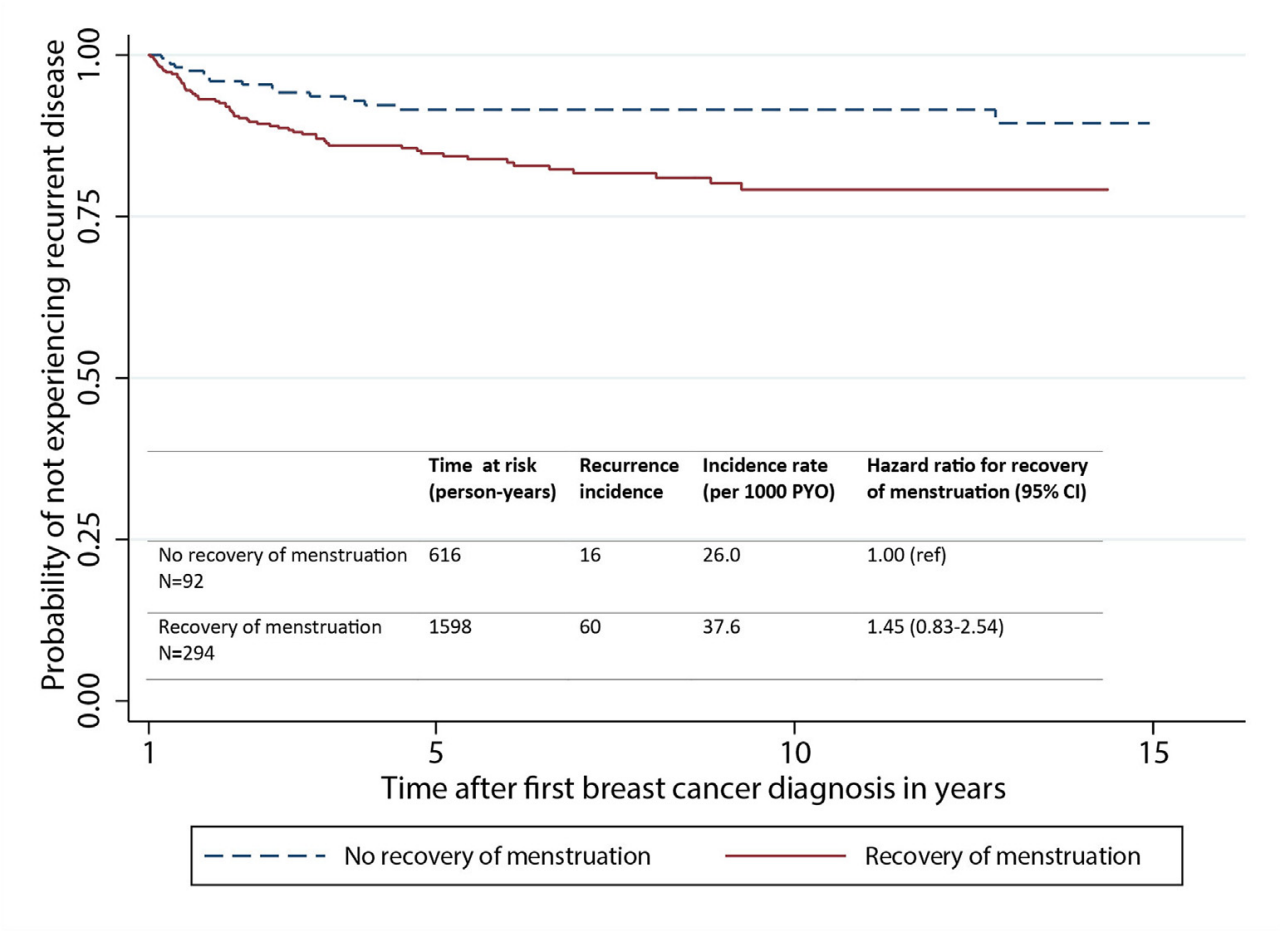

Fig. 2B. Overall survival.

**Figure:**