# Supplementary material for: The impact of menstruation persistence or recovery after chemotherapy on survival in young patients with hormone receptor negative breast cancer
Source: Breast. 2020 May 22;52:102–9. doi: 10.1016/j.breast.2020.05.004 (PMC7375582; doi:10.1016/j.breast.2020.05.004)
Supplement: Multimedia component 1 [file mmc1.docx]

|  | | |  |
| --- | --- | --- | --- |
| Analysis | **Endpoint events** | **Censoring events** | **Hazard ratio for recovery of menstruation compared to no recovery after chemotherapy^‡^** |
| Primary disease-free survival analysis, n=386 | Loco-regional recurrence  Distant metastases  BC-related death  Ipsilateral second breast cancer | Any new cancer diagnosis* (except ipsilateral second breast cancer), non-BC related death, end of follow-up. | Univariable HR: 1.24 (0.71-2.15)  Multivariable HR: 1.45 (0.83-2.54)  Adjusted for: Lymph-node status  Full model HR: 1.31 (0.66-2.59) |
| Disease-free survival sensitivity analysis 1, n=386 | Loco-regional recurrence  Distant metastases  BC-related death  Ipsilateral second breast cancer  Contralateral breast cancer | Any new cancer diagnosis* (except ipsilateral second breast cancer, contralateral breast cancer), non-BC related death, end of follow-up. | Univariable HR: 0.99 (0.67-1.46)  Multivariable HR: 1.12 (0.75-1.68)  Adjusted for: Lymph-node status  Full model HR: 1.15 (0.69-1.92) |
| Disease-free survival sensitivity analysis 2, n=386 | Loco-regional recurrence  Distant metastases  BC-related death | Any new cancer diagnosis*, non-BC related death, end of follow-up. | Univariable HR: 1.17 (0.66-2.08)  Multivariable HR: 1.39 (0.78-2.49)  Adjusted for: Lymph-node status  Full model HR: 1.29 (0.64-2.63) |
| Overall survival Analysis, n=397 | Death from any cause | End of follow-up. | Univariable HR: 1.07 (0.66-1.76)  Multivariable HR: 1.19 (0.71-1.98)  Adjusted for: Lymph-node status, endocrine therapy  Full model HR: 0.98 (0.51-1.88) |

**^Supplementary Table A.1. Overview of all analyses, including sensitivity analyses^**^. Abbreviations: BC, breast cancer; HR, hazard ratio.^

^* Incidental FIGO stage 1 ovarian carcinoma, cervical intra-epithelial neoplasia and non-melanoma skin cancer are not censored for.^

^‡ Multivariable hazard ratio is for the model constructed using the forward stepwise process described in the Methods section. The full model includes all clinically relevant variables, regardless of statistical significance (i.e. age, tumor size, lymph node status,^ *^BRCA^* ^mutation, risk-reducing salpingo-oophorectomy, neoadjuvant therapy, endocrine therapy).^

**Supplementary fig. A.1. Pooled analysis of our study with Park et al. 2012 [16].**

**Supplementary fig. A.2. Kaplan-Meier overall survival curves comparing included to excluded patients. Logrank test: p=0.01.**

|  | | | |
| --- | --- | --- | --- |
|  | **Known menstruation**  N= 417^1^ (66%) | **unknown menstruation**  N= 214^2^ (34%) | **p-value** |
| Follow-up time (at risk) in years, median (range) | 6.3 (1.0 – 27.0) | 5.4 (1.0 – 19.4) | 0.0278 |
| Age at diagnosis, median years (range) | 36.8 (23.6 - 49.9) | 40.0 (24.7 – 49.8) | <0.001 |
| Year of diagnosis, median (range) | 2004 (1990-2014) | 2007 (1991-2014) | <0.001 |
| Proven *BRCA* mutation  No  Yes  *BRCA1*  *BRCA2*  *BRCA1+BRCA2* | 228 (55)  189 (45)  172 (41)  16 (4)  1 (0) | 182 (85)  32 (15)  32 (15)  0  0 | <0.001 |
| Tumor size (pT/cT) ^†^  1  2  3  4  Unknown | 177 (43)  189 (46)  27 (6)  19 (5)  5 | 69 (33)  114 (54)  16 (7)  13 (6)  2 | 0.090 |
| Lymph-node status (pN/cN) ^†^  0  1  2  3 | 204 (49)  135 (32)  43 (10)  35 (8) | 113 (53)  54 (25)  27 (13)  20 (9) | 0.301 |
| Tumor grade  <3  3  Unknown | 37 (10)  346 (90)  34 | 15 (8)  183 (92)  16 | 0.606 |
| HER2 status  HER2+  HER2-  Unknown | 61 (19)  259 (81)  97 | 50 (30)  118 (70)  46 | 0.007 |
|  |  |  |  |
| Radiotherapy received | 296 (71) | 182 (87) | <0.001 |
| Neoadjuvant chemotherapy | 49 (12) | 33 (16) | 0.172 |
| Endocrine therapy | 23 (6) | 0 | <0.001 |
| Targeted therapy | 45 (11) | 37 (17) | 0.018 |

**^Supplementary table A.2. Patient and tumor characteristics of patients with known and unknown menstruation.^**

^1. Two patients died or were lost to follow-up within the first year after diagnosis, and are excluded from the analyses.^

^2. In the group of patients with insufficient data on menstruation for inclusion, five died or were lost to follow-up within one year of diagnosis and are therefore excluded from the analyses.^

^† For patients who were treated with neoadjuvant chemotherapy, the clinical stage is reported.^
